# ZNF146/OZF and ZNF507 target LINE-1 sequences

**DOI:** 10.1093/g3journal/jkac002

**Published:** 2022-01-17

**Authors:** Kevin M Creamer, Eric C Larsen, Jeanne B Lawrence

**Affiliations:** Department of Neurology and Pediatrics, University of Massachusetts Medical School, Worcester, MA 01655, USA

**Keywords:** ZNF146, OZF, ZNF507, PRMT5, LINE-1, L1, transposable element

## Abstract

Repetitive sequences including transposable elements and transposon-derived fragments account for nearly half of the human genome. While transposition-competent transposable elements must be repressed to maintain genomic stability, mutated and fragmented transposable elements comprising the bulk of repetitive sequences can also contribute to regulation of host gene expression and broader genome organization. Here, we analyzed published ChIP-seq data sets to identify proteins broadly enriched on transposable elements in the human genome. We show 2 of the proteins identified, C2H2 zinc finger-containing proteins ZNF146 (also known as OZF) and ZNF507, are targeted to distinct sites within LINE-1 ORF2 at thousands of locations in the genome. ZNF146 binding sites are found at old and young LINE-1 elements. In contrast, ZNF507 preferentially binds at young LINE-1 sequences correlated to sequence changes in LINE-1 elements at ZNF507’s binding site. To gain further insight into ZNF146 and ZNF507 function, we disrupt their expression in HEK293 cells using CRISPR/Cas9 and perform RNA sequencing, finding modest gene expression changes in cells where ZNF507 has been disrupted. We further identify a physical interaction between ZNF507 and PRMT5, suggesting ZNF507 may target arginine methylation activity to LINE-1 sequences.

## Introduction

In the wake of whole-genome sequencing, it has become clear that only a small portion of DNA codes for protein in higher organisms. While only about 1–2% of the roughly 3 billion bases in the human genome are protein-coding, an astounding 40–50% is derived from mobile repetitive sequences collectively known as transposable elements (TEs). Mobilization of TEs, sometimes referred to as “jumping genes,” poses a threat to host genome stability and is known to sometimes cause disease (Kazazian and Moran 2017). In recent years, however, TEs and TE-derived sequences have been implicated as drivers of gene expression patterning, genome organization, development, and evolution in higher organisms (Rebollo *et al.* 2012; Hall *et al.* 2014; Lu *et al.* 2021).

TEs multiply and spread in host genomes by “cut-and-paste” transposition of their DNA sequence or through an RNA intermediate (retrotransposition). Ultimately, to preserve genomic integrity TEs are largely inactivated by various mechanisms encoded by the host genome. Relatively few mobile TEs persist in the human genome, primarily consisting of a hundred or so long interspersed nuclear elements (LINE-1 or L1) and SVA (short interspersed nuclear element–VNTR–Alu) repeats that require L1 encoded reverse transcriptase activity to mobilize (Brouha *et al.* 2003; Wang *et al.* 2005). Instead, most TE DNA in the genome consists of degenerate remnants of prior mobilization events that have been truncated or mutated and no longer harbor the activity necessary to “jump.”

Transposition-competent TEs are recognized by DNA-binding proteins and generally inactivated at the level of transcription, perhaps most notably by KRAB-containing zinc finger proteins that recruit repressive epigenetic modifications to chromatin and by DNA methylation (Yang and Wang 2016; Yang *et al.* 2017). TEs sometimes, however, escape this repression. In humans, L1s mobilize in the germline and are a major source of inheritable structural variation between individuals (Ewing and Kazazian 2010; Mir *et al.* 2015). Recent studies have revealed that L1 elements are also activated in specific contexts outside of the germline, such as stress, cancer, and neural development, with disease-amplifying or functional implications (Coufal *et al.* 2009; Burns 2017; Faulkner and Garcia-Perez 2017; Kazazian and Moran 2017).

Irrespective of their mobility, abundant TE sequences are nonrandomly distributed in host genomes and contribute to development and gene expression patterning through recognition by DNA-binding proteins (Manuelidis and Ward 1984; Korenberg and Rykowski 1988; Bourque *et al.* 2008; Rebollo *et al.* 2012). For instance, HERV-H/MERV-L TEs are known to be expressed early during embryogenesis and are important regulators of pluripotency and embryogenesis by regulating the expression of neighboring genes, a process driven by sequence-specific recognition of TE DNA by transcription factors (TFs) (Kigami *et al.* 2003; Wang *et al.* 2014; Robbez-Masson and Rowe 2015). L1 elements are also known to be highly expressed in the early mouse embryo and contribute to proper embryonic development (Fadloun *et al.* 2013; Jachowicz *et al.* 2017).

While several DNA-binding proteins are known to target TEs, the complex picture of TF binding to TE sequences remains incomplete and the functional significance of these interactions on cell or organism biology is understudied, and largely unknown. Here, we analyze published ChIP-seq data sets to identify proteins that broadly recognize TE-derived sequences in the human genome. We focus on 2 poorly studied zinc finger proteins, ZNF146 (also known as OZF) and ZNF507, and characterize their recognition motifs within L1 repeats. Interestingly, we find ZNF146 is a highly conserved protein that retains targeting to thousands of relatively old L1 sequences in the genome. In contrast, ZNF507 was only observed at relatively young L1 sequences. We go on to functionally test the impact depletion of these proteins has on the transcriptome of HEK293 cells. Finally, we identify an interaction between ZNF507 and PRMT5 by co-immunoprecipitation and mass spectrometry, yielding potential insight into the function of ZNF507 in the cell.

## Materials and methods

### TE enrichment analysis

All TF ChIP-seq experiments with a corresponding IDR threshold peak file were downloaded from ENCODE in March 2020 (hg19 only). For each experiment, peaks were shuffled 10 times using BEDtools shuffle excluding ENCODE blacklist regions and -noOverlapping -maxTries 1000 parameters (Quinlan and Hall 2010). Frequency of intersection between experimental or shuffled peaks and Repeatmasker repeat classes were calculated using BEDtools intersect and the -u parameter. Heatmaps were graphed in R.

### LINE-1 enrichment analysis

Meta-analysis of ChIP enrichment at LINE-1 elements was performed using unique reads (fold change over control bigwig files downloaded from ENCODE). Primary alignments were also downloaded from ENCODE and coverage files of fold change over control coverage were also generated without filtering for uniqueness using deepTools bamCoverage and the –normalizeTo1X parameters (Ramirez *et al.* 2014). Meta-analysis was performed at full-length L1 elements annotated by L1Base or binned by subfamily from Repeatmasker (Penzkofer *et al.* 2017). Mappability tracks were downloaded from the UCSC table browser. Heatmaps were generated using deepTools computeMatrix and plotHeatmap software.

Coverage of peaks aligned to the L1PA1 consensus was performed by extracting reads (ENCODE) mapping to L1 elements using BEDtools intersect. Aligned sequences were then retrieved using BEDtools getfasta. Fasta sequences were then directly aligned to the L1PA1 consensus sequence using bwa mem and the -B 1 -O 1 -d 1 -T 1 -r 0.1 -t 12 -k 10 parameters (Li and Durbin 2009). Coverage was then calculated using Samtools depth. Signal over input was then calculated and graphed in R using ggplot2.

### Motif discovery and scanning

Motif discovery was performed on ENCODE peak DNA sequences using MEME software and order-0 background, classic discovery mode, 0 or 1 occurrence, 13mer parameters (Bailey and Elkan 1994). Motif occurrences in the genome were found using the top identified motif for each ChIP experiment and FIMO software, filtered by the indicated *P*-value cutoff (Grant *et al.* 2011).

### Zinc finger identification and multiple sequence alignments

Protein sequences were downloaded for alignment from UniProt. L1 sequences were downloaded from Dfam. Zinc fingers were predicted and annotated using online software (http://zf.princeton.edu/, last accessed September 2021) (Persikov *et al.* 2009). Multiple sequence alignments were performed using ClustalX and Jalview with additional manual curation to focus on zinc finger residues and presentation.

### CRISPR/Cas9 disruption and RNA sequencing

HEK293 cells were grown in DMEM (Thermo Fisher) media and transiently transfected with equal amounts of Cas9 and sgRNA expressing plasmids (PX459) targeting either ZNF146, ZNF507 or an equivalent amount of empty vector control plasmid (Ran *et al.* 2013). Target sequences used for ZNF146 were 5′-ACTGAGCATGAGCATTTTC-3′, 5′-ACATGTACAATAAGTGATG-3′, 5′-ACTGTAAATTCTCTGCTGGC-3′, and 5′-TGAAGGTTTTTCCACACTC-3′. Target sequences used for ZNF507 were 5′-TGGGCTTCAAGTTCCTCC-3′, 5′-ATTGTTTCCGGACAAACTT-3′, 5′-CTGGCTTCTAGATGTAATA-3′, and 5′-AGGTTGGCTCTTGTCAACTC-3′. The day after transfection cells were selected for 36 h using puromycin before outgrowth for a total of 2 weeks prior to analysis.

Western blots were performed using antibodies against ZNF146 (Novus Biologicals) or ZNF507 (Thermo Fisher). RNA was isolated from cells disrupted with CRISPR/Cas9 in biological duplicate using Trizol RNA extraction reagent. RNA was treated with TURBO DNase (Thermo Fisher) to remove contaminating DNA and cleaned up using RNeasy Mini (Qiagen) kits according to manufacturer recommendations. RNA sequencing libraries were prepared using a stand-specific kit RNA-seq kit with ribosome depletion (KAPA Biosystems) and NEBNext Adapters and Multiplex Oligos for Illumina. Paired-end 50 bp sequencing was performed on the Illumina HiSeq 2000 platform (UMMS Core Facilities).

Reads were pre-processed and clipped using the Fastx Toolkit and those mapping to ribosomal RNA were removed prior to subsequent mapping with Bowtie. Nonribosomal reads were then mapped to the human genome (hg19) using TopHat2 software with –library-type fr-firststrand –no-coverage-search parameters and Ensembl (ver82) gene model annotations. Differential expression analysis was performed using count matrices generated by featureCounts and DeSeq2 (Liao *et al.* 2014). Associated graphs were generated using ggplot2 within R. The associated fastq files and count matrices for this experiment are available at GEO: GSE172285.

### Expression, immunofluorescence analysis, and immunoprecipitation of FLAG-tagged ZNF507

To express 3xFLAG-tagged ZNF507, RNA ZNF507 cDNA was amplified from HeLa cell RNA using NEBNext High-Fidelity 2X PCR Master Mix and the following oligos: 5′-GATCGATCGGATCCGAAGAAAGTAGCAGTGTTGCCATGTTGGTG-3′ and 5′-GATCGATCGAATTCCTAATTTGTGTTTAGAGCTGTATTGTGGTCCTTATTCAGG-3′. ZNF507 cDNA was then and cloned into a pcDNA3.1 n-terminal 3xFLAG expression vector (CMV promoter).

For immunofluorescence analysis, HEK293 cells were grown on coverslips in 6 well dishes and transfected with pcDNA3.1-3XFLAG-ZNF507 plasmid using lipofectamine reagent (Thermo Fisher). After 36 h cells were fixed with 4% paraformaldehyde in PBS for 10 min at room temperature. Coverslips were then incubated with a 1:100 dilution of M2 anti-FLAG antibody (Sigma) in 1% BSA, 1X PBS at 37°C for 1 h, then washed, and immunodetected using 1:500 dilution of conjugated secondary antibody, in 1X PBS with 1% BSA. Nuclei were visualized using Axiovert 200 microscope equipped with a 100X PlanApo objective (NA 1.4) and Chroma 83000 multibandpass dichroic and emission filter set (Bratteboro). Images were captured using a cooled charge-coupled device (CCD) camera (200 series, Photometrics). Images were minimally corrected for brightness and contrast using standard practices to best represent signals observed by eye using Zen (Zeiss) software. In cells with signal, 3xFLAG-ZNF507 consistently was localized to the nucleus. No appreciable signal was observed for cells transfected with empty vector control.

Immunoprecipitation of 3xFLAG-ZNF507 and associated proteins was performed by transfecting approximately 40 million HEK293 cells with either 3x-FLAG-ZNF507 expression plasmid or an empty vector control. After 36 h, cells were dislodged from flasks in ice cold 1X PBS and harvested in 10 ml falcon tubes. Cells were washed twice in ice cold PBS before lysis for 5 min on ice in 1 ml Lysis Buffer [10 mM HEPES pH 7.5, 1.5 mM MgCl2, 10 mM NaCl, 0.075% NP-40, 1 mM PMSF, 1X EDTA-free protease inhibitor cocktail (Roche)]. Crude nuclear pellets were then harvested by centrifugation at 4°C for 3 min at 1,000 × g and removal of supernatant. Nuclei were then suspended in 1 ml Nuclear Extraction Buffer [20 mM Tris-HCl pH7.5, 500 mM NaCl, 1.5 mM MgCl2, 25% glycerol, 1 mM PMSF, 1X EDTA-free protease inhibitor cocktail (Roche)], vortexed briefly, and incubated 15 min on ice. Insoluble DNA and debris were then cleared from the nuclear extract by centrifugation at 4°C for 10 min at 12,000 × g. To the supernatant an equal amount of Dilution buffer [20 mM Tris-HCl pH 7.5, 1 mM EDTA, 0.2% Triton-X 100, 1 mM PMSF, 1X EDTA-free protease inhibitor cocktail (Roche)] was added to lower salt concentration before immunoprecipitation.

Nuclear extracts were pre-cleared by incubation with 40 µl Protein A dynabeads (Sigma) for 1 h at 4°C. After capture of beads, 40 µl anti-FLAG M2 magnetic beads (Sigma) were added to each nuclear extract and incubated 6 h at 4°C. Beads and co-immunoprecipitated proteins were then captured and washed twice for 5 min with rotation at 4°C with Wash buffer (20 mM Tris-HCl pH 7.5, 150 mM NaCl) containing 0.1% Triton X-100 and once without detergent. Proteins were eluted twice using 50 µl of 3X-FLAG peptide at a concentration of 500 ng/µl and incubated with rotation at 4°C for 30 min for each elution.

Laemmli sample buffer (4X) was added to each elution to 1X final concentration and samples were incubated for 15 min at 80°C. Samples were then briefly run on the same 4%–20% Criterion Stain Free Tris-HCl Protein Gel separated by several lanes so that the dye front was 2 cm below each well. Lanes were cut out slightly above the dye to the well bottom for LC-MS/MS analysis (UMMS Mass Spectrometry Core Facility). Mascot was set up to search SwissProt_Human and Scaffold (Proteome Software Inc.) was used to validate MS/MS based peptide and protein identifications.

## Results

### Identification of DNA-binding proteins that target TEs

**Fig. 1.**
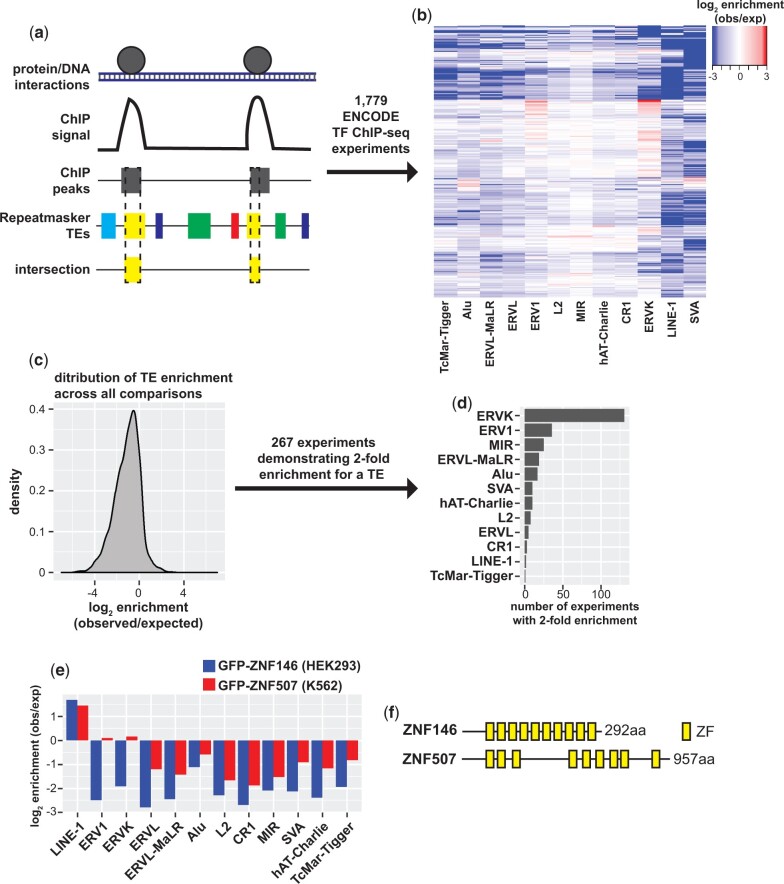
Identification of TFs that bind transposable elements. a) Schematic of ENCODE TF ChIP-seq peak intersection with Repeatmasker annotated TEs. b) Heatmap of TF ChIP-seq enrichment on abundant TE classes calculated as the log_2_ ratio of observed (obs) peak/TE intersections relative to simulated shuffled peaks (expected, exp). c) Density of enrichment across all ChIP-seq experiment peak and TE class comparisons. d) Bar chart of the number of TF ChIP-seq experiments demonstrating 2-fold enrichment binned by TE class. e) Bar chart of peak enrichment on TE classes for GFP-ZNF146 ChIP-seq in HEK293 cells and GFP-ZNF507 in K562 cells. f) Schematic representation of ZNF146 and ZNF507 proteins and the distribution of their C2H2 zinc finger domains.

In order to identify proteins that broadly target TEs we developed a strategy to test chromatin immunoprecipitation with sequencing (ChIP-seq) data sets for enrichment on TEs using a simple peak intersection strategy (Fig. 1a). For this study, we analyzed all ENCODE transcription factor (TF) ChIP-seq experiments (as of March 2020, see Supplementary Table 1) for which peaks had been identified, spanning a large number of transcription factors, cell types, and sequencing methodologies (ENCODE Project Consortium 2012; Imbeault *et al.* 2017). Peaks were then intersected with Repeatmasker annotated repeats for the most abundant TE classes in the human genome. Frequency of intersection between peaks for each experiment was then compared to frequency of intersection with background models generated by random shuffling to generate a ratio of observed elements relative to what would be expected by random chance (Fig. 1b).

While most peak/TE comparisons indicated TF binding to TEs occurred less frequently than expected by random chance (Fig. 1c), 361 experiments indicated peak enrichment of greater than 2-fold (observed/expected), 267 of which were enriched for a single TE class (Fig. 1d). Interestingly, enrichment was most frequently (>100 experiments) observed for ERVK transposons. We were additionally struck with the lack of TFs identified by this approach targeting LINE-1 elements. Just 2 experiments, ChIP-seq for GFP-ZNF146 in HEK293 and GFP-ZNF507 in A529 cells demonstrated 2-fold enrichment for LINE-1 elements. This enrichment was specific for LINE-1 as these proteins were not associated with other TE classes (Fig. 1e).

ZNF146 (also known as OZF or Only Zinc Fingers) and ZNF507 genes are both found on chromosome 19 and encode proteins with 10 and 9 C2H2 zinc finger motifs. Neither protein has any other easily identifiable domains (Fig. 1f), and apparently lack KRAB domains frequently found in zinc finger proteins known to silence TEs (Yang *et al.* 2017). Zinc finger motifs can contribute to protein–protein and protein–RNA interactions, but are best studied for their contribution to transcription factor binding to specific DNA sequences (Laity *et al.* 2001). ZNF146/OZF was previously identified as being overexpressed in certain cancers (Ferbus *et al.* 1999, 2003). Still, neither ZNF146 or ZNF507 have been extensively studied and little is known about their function despite being predicted to be widely expressed in human cells (Uhlen *et al.* 2015, 2017).

LINE-1 sequences are widely distributed and highly abundant in the human genome. Most L1 sequences are truncated and degenerate, with varying divergence from transposon-competent consensus sequences. There are estimated to be just 100 transposition-competent L1 elements, 4,000 mutated but full-length elements, and several hundred thousand truncated L1 fragments in the human genome (Brouha *et al.* 2003). Because of LINE-1 prevalence in the human genome, the small number and specificity of TFs identified by this approach, and the understudied nature of the 2 proteins identified, we decided to characterize the targeting of ZNF146 and ZNF507 to L1 elements in greater detail.

### ZNF146 and ZNF507 target LINE-1 ORF2

**Fig. 2.**
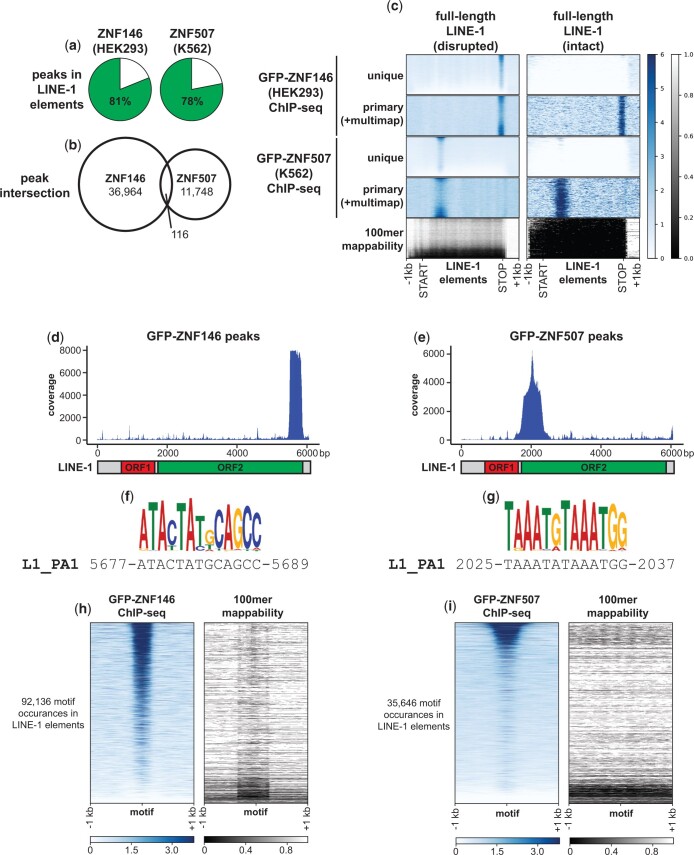
ZNF146 and ZNF507 target LINE-1 sequences. a) Pie chart representing the percentage of peaks in the indicated ChIP-seq experiments overlapping annotated LINE-1 elements. b) Venn diagram of peak interval intersections between the indicated ChIP-seq experiments. c) Meta-analysis of ChIP-seq enrichment at full-length disrupted (*n* = 13,092) or intact (ORF2 or ORF1 and ORF2) LINE-1 elements (*n* = 245) normalized to 5 kb. Below is the 100mer mappability score for the same regions (lower scores indicate lower mappability) d, e) Coverage of L1-mapping reads directly mapped to LINE-1 (human specific, PA1) consensus sequence. Below is a schematic of full-length LINE-1. f, g) Enriched motifs (see *Materials and Methods*) and corresponding LINE-1 (human specific, PA1) consensus sequence determined from ZNF146 and ZNF507 ChIP-seq experiments, respectively. h, i) Heatmap of ChIP-seq enrichment centered around occurrences of identified motifs (FIMO, *P* < 5e-6) found in annotated LINE-1 elements in their genomic context. On the right is the theoretical mappability of 100 bp reads in the same regions.

The above analyses indicated ZNF146 and ZNF507 preferentially bind to LINE-1 sequences. In order to verify these observations, we performed additional in-depth analysis of ENCODE’s ChIP-seq experiments. Consistent with L1 targeting, peaks for both ZNF146 and ZNF507 ChIP-seq experiments were found mostly (81% and 76% of peaks, respectively) within annotated L1 elements (Fig. 2a). Despite high frequency of targeting to L1 elements for both proteins, ZNF146 peaks and ZNF507 peaks themselves did not frequently coincide (Fig. 2b), suggesting they target different sequences within L1 elements.

To test this idea we performed meta-analysis of ChIP-seq coverage for both proteins at full-length L1 elements (Fig. 2c). Due to their repetitive nature, full-length L1 elements are often difficult to analyze by sequencing methods, however for these experiments ENCODE performed 100 bp paired-end sequencing, allowing for relatively high mappability at disrupted (degenerate or mutated), full-length elements (Fig. 2c, left panels). In contrast, intact (retaining coding potential) L1s had very low mappability (Fig. 2c, right panels).

Using uniquely mapping reads, concentrated enrichment of ChIP-seq signal was observed for both ZNF146 and ZNF507 at disrupted, full-length L1s, with ZNF507 enrichment most prominent toward the 5’ end and ZNF146 toward the 3’ end of these elements. Using primary reads (which allow for multimapping reads to be randomly assigned to 1 location), similar enrichments could be observed on intact L1 elements. Hence while it is not possible to ascertain whether ZNF146 and ZNF507 bind at individual, intact elements, these proteins likely target intact L1s as well.

We then resolved ZNF146 and ZNF507 binding by calculating coverage after performing local alignment of peak sequences to the L1PA1 (human specific) consensus sequence. LINE-1 transposons code for 2 proteins, ORF1p and ORF2p, both of which are required for transposition of LINE-1 (Moran *et al.* 1996). ORF1 encodes a chaperone while ORF2 encodes a reverse transcriptase essential for transposition of not only L1, but other nonautonomous TEs as well (Mathias *et al.* 1991; Kolosha and Martin 1997; Dewannieux *et al.* 2003; Ostertag *et al.* 2003). ZNF146 and ZNF507 peaks specifically aligned to the 3’ and 5’ ends of the ORF2 coding region of L1, respectively, with no enrichment within ORF1 (Fig. 2, d and e).

We further identified likely binding motifs within L1 ORF2 by performing motif discovery (Fig. 2, f and g). The most significant motif identified for each ChIP-seq experiment closely matched sequences in the L1PA1 consensus sequence at positions of highest ChIP-seq enrichment (Fig. 2, d–g). We then located all instances of the determined motifs for ZNF146 and ZNF507 in L1 elements genome-wide and performed meta-analysis of ChIP-seq signal at these motifs. As expected, robust ChIP-seq signal was found centered around these motifs for both proteins (Fig. 2, h and i). Thus, targeting of ZNF146 and ZNF507 to L1 elements appears to be genuine and occurs at opposite ends of the ORF2 coding region.

### ZNF507 preferentially binds at young LINE-1 sequences

While ChIP-seq signal enrichment was observed for ZNF146 at nearly all mappable instances of its predicted binding sites (Fig. 2h), ZNF507 ChIP-seq signal was enriched at a fraction of its motif sites, suggesting that ZNF507 may only bind to a subset of L1 elements. LINE-1 sequences in the genome have been classified based on diagnostic sequence variants. Comparative genomics and expected sequence divergence over time have further allowed for approximate aging of L1 subfamilies containing these shared sequence variants from youngest (L1PA1, human specific) to oldest (L1PA17) primate-specific LINE-1 elements (Smit *et al.* 1995; Khan *et al.* 2006; Giordano *et al.* 2007; Konkel *et al.* 2010).

**Fig. 3.**
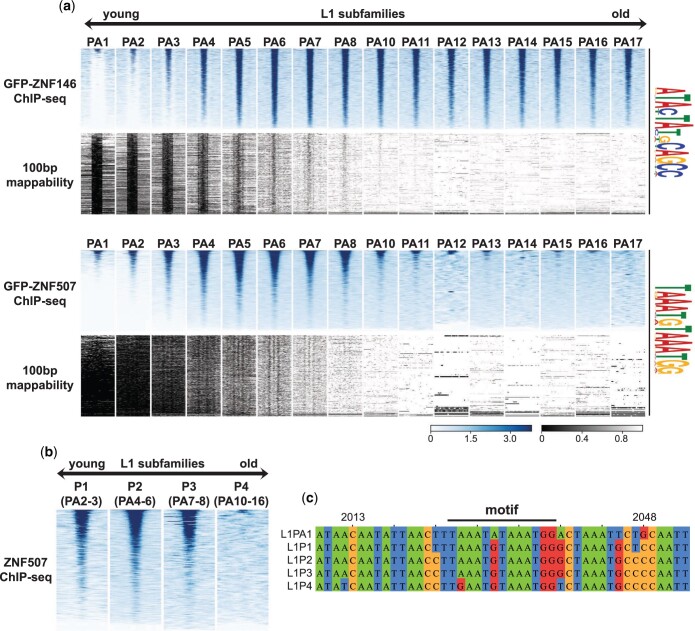
ZNF507 does not bind at old L1PA elements. a) Heatmap of ChIP-seq enrichment centered around occurrences of identified motifs (FIMO, *P* < 5e-6) ± 1 Kb after intersection and binning by LINE-1 subfamily. Below the ChIP-seq heatmaps (blue) is the theoretical mappability of 100 bp reads for the same regions (grayscale). b) Heatmaps as above but for LINE-1 subfamilies binned by their ORF2-derived classification. Relationship between the ORF2 and 3’UTR classifications is given in parentheses. c) Multiple sequence alignment of L1 subfamily consensus sequences. Positions are derived from the L1PA1 consensus sequence. Position of the ZNF507 ChIP seq binding motif identified by MEME is indicated above the alignment.

To determine whether ZNF146 or ZNF507 might differentially recognize L1 subfamilies we binned motif instances in LINE-1 elements by subfamily, and performed meta-analysis of ChIP-seq signals around these motifs. As expected from unbinned meta-analysis (Fig. 2h), ZNF146 ChIP-seq enrichment was observed at nearly all predicted mappable binding sites in all subfamilies (Fig. 3a, upper panels). Because the youngest subfamilies (L1PA1 and L1PA2) are less uniquely mappable, binding to those families could not be reliably determined. We note, however, that those families contain the same conserved binding motif and enrichment can be observed at young, intact elements when including multimapping reads (Fig. 2c), suggesting ZNF146 likely binds at these sequences as well.

In contrast, we observed strong enrichment of ZNF507 only in young subfamilies, particularly in L1PA3-L1PA8 (Fig. 3a, lower panels). Again, while binding at L1PA1 and L1PA2 sequences could not be definitively determined due to mappability, binding of ZNF507 was observed at intact L1 sequences when allowing for multimapping (Fig. 2c), suggesting ZNF507 likely binds at these elements as well. Enrichment was essentially absent or rarely observed for ZNF507 in older subfamilies, particularly PA12-PA17. L1 classifications are determined largely by 3’ UTR sequence variations (as above) but are sometimes classified using upstream ORF2 sequences (L1P1, youngest, to L1P4, oldest). ZNF507 enrichment was clearly observed at motifs in L1P1, L1P2, and L1P3 subfamilies, but essentially absent at motifs in L1P4 elements (Fig. 3b). Interestingly the L1P4 consensus sequence deviates from younger subfamilies within the core motif we identified as well as 2 other nearby residues, perhaps explaining the lack of binding observed for ZNF507 at these elements (Fig. 3c).

**Fig. 4.**
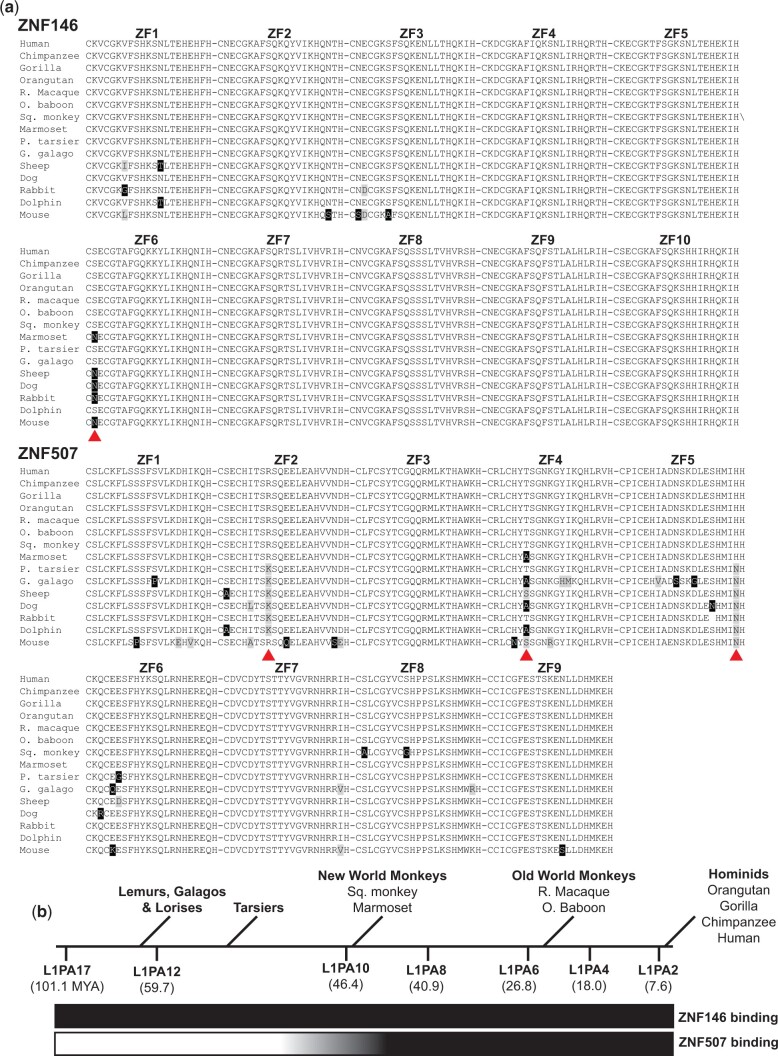
Conservation of ZNF146 and ZNF507 zinc finger domains. a) Alignment of protein sequences for ZNF146 and ZNF507 zinc finder domains (indicated by number above alignment) in candidate mammals. Abbreviated common names are used (R. macaque, Rhesus macaque; O. baboon, Olive baboon; Sq. monkey, Squirrel monkey; P. tarsier, Philippine tarsier; G. galago, Garnett’s galago). Black boxes indicate residue differences from human proteins. Gray boxes indicate nonidentical but similar residues. Red arrows indicate residues conserved in monkeys and apes, but not frequently observed in tarsiers, lower primates, or other mammals. b) Approximate timeline of primate evolution relative to the age of L1PA subfamilies (millions of years ago, MYA), adapted from Khan *et al.* and Konkel *et al.* Qualitative assessment of ZNF146/ZNF507 binding to L1 subfamilies (inferred from ChIP-seq) is displayed as a gradient below from nonbinding (white) to binding (dark).

Zinc finger proteins and TEs are in some cases thought to have co-evolved in an “evolutionary arms race” of natural selection (Jacobs *et al.* 2014; Cosby *et al.* 2019). Since ZNF507 binding was observed only at young L1 subfamilies, we wondered if this specificity change could be related to amino acid substitutions in ZNF507 zinc fingers. Interestingly, multiple sequence alignment of ZNF507 ZFs revealed 3 separate residues in ZNF507 ZFs that are highly conserved among higher primates (apes and monkeys) but were not found in lower primates and other mammals (Fig. 4a). The timing of these substitutions appeared to closely parallel changes in L1 sequence and differences in ZNF507 binding (compare timeline in Fig. 4b to Fig. 3, a–c), collectively suggesting a relationship between the amino acid substitutions in ZNF507 ZFs and L1 targeting specificity.

### Impact of ZNF146 and ZNF507 depletion on a somatic cell transcriptome

The above results demonstrate ZNF146 and ZNF507 target thousands of L1 sequences in the genome. Binding at these regions could potentially influence L1 or endogenous gene expression. We therefore disrupted ZNF146 and ZNF507 expression in human embryonic kidney cells (HEK293) using CRISPR/Cas9 to determine what impact depleting these proteins can have on the transcriptome of a somatic cell line.

**Fig. 5.**
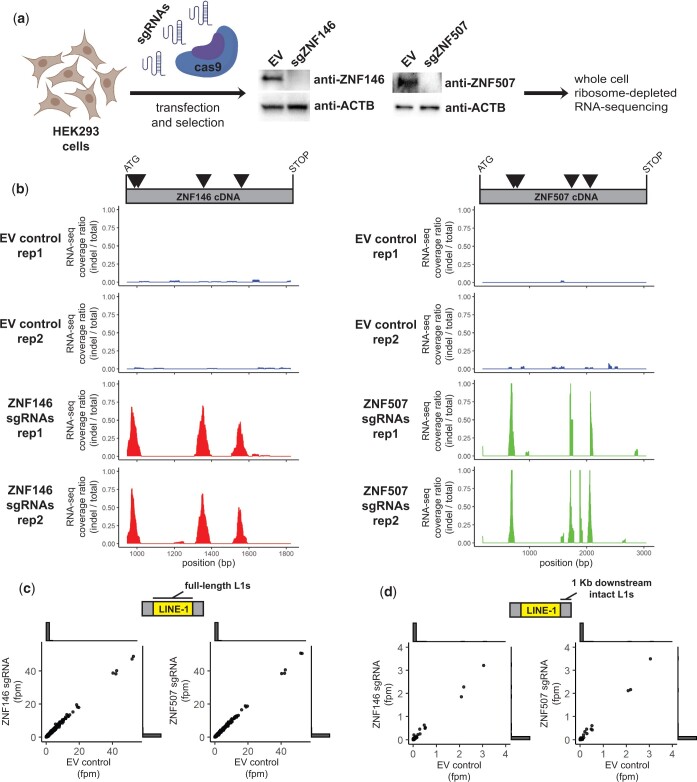
ZNF146 and ZNF507 are not required to silence LINE-1 elements in HEK293 cells. a) Schematic of CRISPR/cas9 disruption approach. HEK293 cells were transiently transfected with spCas9-sgRNA-puro plasmids with either sgRNAs targeting ZNF146 or ZNF507 respectively or an empty vector (EV) control. Cells were briefly selected with puromycin before outgrowth. Fourteen days post-transfection cells were harvested for western blot analysis (middle panels) to assess disruption of protein expression. RNA was isolated for RNA-sequencing analysis. Disruption and RNA-sequencing were performed in duplicate. b) RNA-sequencing coverage was calculated for reads mapping to ZNF146 (left) or ZNF507 cDNA (right) after filtering for reads marked with or without indels during alignment. Data is presented as the ratio of coverage calculated for reads with indels (determined by CIGAR tags) over total reads in each RNA-sequencing replicate. Triangles in the schematic above represent sgRNA targets. c) Comparison of RNA-sequencing depth in fragments per million (fpm) mapping to individual full-length LINE-1 elements. Outside of the scatter plot are histograms showing most values are at or near zero. d) Comparison of RNA-sequencing depth in fragments per million (fpm) mapping to 1 Kb regions downstream of intact (ORF1/ORF2 or ORF2 only) LINE-1 elements. Outside of the scatter plot are histograms indicating most values are at or near zero. Schematics created with BioRender.com.

Cells were transiently transfected with Cas9 and sgRNA expressing plasmids targeting either gene or a nonempty vector control (Ran *et al.* 2013). After transient selection and outgrowth, whole-cell extracts were analyzed by western blot to confirm target gene disruption. Figure 5a shows ZNF146 and ZNF507 were essentially undetectable in Cas9-targeted cells (Fig. 5a), which had no obvious growth or morphology defects. We then isolated RNA and performed transcriptomic analysis by paired-end RNA sequencing following ribosome depletion.

First, we confirmed Cas9 targeting by aligning RNA-seq reads to ZNF146 and ZNF507 cDNA reference sequences using Tophat2 and BWA aligners to rescue unmapped reads as previously described (Deininger *et al.* 2017). We then compared the coverage ratio of reads marked as having insertions or deletions (indels) during alignment *vs.* reads without indels. For both ZNF146 and ZNF507 indels were frequently detected at sgRNA target sites that were not observed in control cells, consistent with efficient disruption and protein depletion observed by western blot (Fig. 5b).

We then assessed whether we could detect an increase in RNA originating from full-length L1 elements. Full-length and intact L1 elements are known to be heavily repressed in most cell lines. Because of this and their repetitive nature, the vast majority of full-length elements were not detected using uniquely mapping RNA-seq reads (see histogram of counts at or near zero in Fig. 5c). For those that were detectable we did not observe any that were differentially expressed (*P* < 0.05) in cell populations in which ZNF146 or ZNF507 were disrupted (Fig. 5c).

Although intact LINE-1 elements are themselves highly unmappable by unique reads, their expression can reliably be detected in RNA-seq experiments by quantifying reads mapping to immediately downstream regions which become expressed as a result of read-through transcription (Philippe *et al.* 2016). Similar to what we observed within L1 elements, few RNA-seq reads mapped downstream of intact L1 elements, consistent with their general repression (Fig. 5d). For those downstream regions for which we did observe low-level expression, transcripts from these regions were not differentially expressed when ZNF146 or ZNF507 were disrupted. Although we cannot rule out increased levels of rare or stochastic L1 activation, these results indicate L1 elements are not broadly activated in response to ZNF146 and ZNF507 depletion in HEK293 cells.

**Fig. 6.**
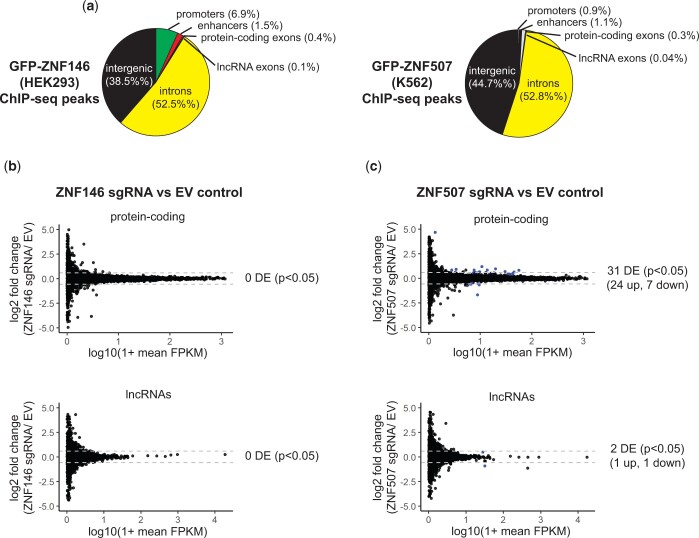
Disruption of ZNF507 leads to modest changes in gene expression. a) Pie chart of ChIP-seq peak intersection with genomic features. Promoters and enhancers are PHAMTOM-annotated. Genic features are RefSeq-annotated. b, c) Comparison of RNA-sequencing depth in fragments per kilobase per million mapped reads (FPKM) mapping to protein-coding transcripts (top) or lncRNAs (bottom). ZNF146 (left) or ZNF507 (right) disrupted cells are compared to EV control. Dotted gray lines mark 1.5-fold change. Transcripts which were differentially expressed (*P* < 0.05) are colored blue.

ZNF146 and ZNF507 ChIP-seq peaks are not restricted to intergenic regions as we also observed many peaks for both proteins in regulatory and genic regions including at annotated enhancers and promoters (Fig. 6a). In addition, a large numbers of peaks (>50%) for both ZNF146 and ZNF507 were located in introns. Targeting to these regulatory and genic regions could potentially influence endogenous gene expression.

We therefore assessed what impact, if any, loss of ZNF146 or ZNF507 had on gene expression. Remarkably, despite observed binding peaks at over 40,000 sites in ChIP-seq data, we did not observe differential expression of any protein-coding or lncRNA (*P* < 0.05) in cells depleted of ZNF146 in HEK293 cells (Fig. 6b). Interestingly, however, ZNF507 disruption resulted in the differential expression of 30 protein-coding (24 up and 6 down-regulated) and 2 lncRNA (1 up and 1 downregulated) transcripts, indicating depletion of ZNF507 influenced nonretroviral gene expression (Fig. 6c, Table 1). Two differentially expressed genes had ZNF507 ChIP-seq peaks within 10 kb of their transcription start sites and twelve genes had peaks located within 100 kb, a common distance cutoff for cis regulatory elements (Vijayabaskar *et al.* 2019), suggesting these genes could potentially be directly regulated by ZNF507.

**Table 1.** Differentially expressed protein-coding genes after ZNF507 disruption.

ENSEMBL ID	Symbol	log2 fold change (sgZNF507/EV)	*P*adj^a^	Dist. to ZNF507 peak (kb)^b^
ENSG00000198715	GLMP	−1.705643082	6.99E-29	384,886
ENSG00000103174	NAGPA	1.169592472	4.40E-22	106,467
ENSG00000110442	COMMD9	0.947934187	5.24E-18	386,015 (266,153)
ENSG00000122965	RBM19	0.689678085	2.15E-16	335,257
ENSG00000059691	GATB	0.821869765	3.65E-16	164,703
ENSG00000102393	GLA	0.81066298	2.52E-15	40,890
ENSG00000185896	LAMP1	0.637840578	7.58E-15	89,326
ENSG00000090581	GNPTG	0.818300413	6.83E-13	57,137
ENSG00000167716	WDR81	−0.690739581	1.18E-10	4,257
ENSG00000124839	RAB17	4.677234569	3.18E-10	256,078
ENSG00000160695	VPS11	0.6614881	1.20E-09	330,782
ENSG00000168813	ZNF507	−0.613938125	2.11E-09	118,073
ENSG00000198908	BHLHB9	−0.758590795	3.57E-08	60,179 (30,831)
ENSG00000103042	SLC38A7	0.611608645	5.06E-08	6,878
ENSG00000176994	SMCR8	0.457690189	2.37E-07	372,506
ENSG00000197081	IGF2R	0.416262343	2.75E-07	26,007
ENSG00000103249	CLCN7	0.457423373	1.32E-06	180,322
ENSG00000157593	SLC35B2	0.494377197	1.43E-06	53,461
ENSG00000106266	SNX8	−0.545579961	1.82E-06	266,887
ENSG00000047249	ATP6V1H	0.570565355	2.76E-06	306,030
ENSG00000064601	CTSA	0.487320189	3.47E-06	87,743
ENSG00000144455	SUMF1	0.836597857	5.32E-06	63,483
ENSG00000159720	ATP6V0D1	0.394188331	5.88E-06	112,601 (58,771)
ENSG00000244045	TMEM199	0.590532873	6.49E-06	54,704
ENSG00000087088	BAX	0.414954399	8.94E-06	624,238
ENSG00000242802	AP5Z1	0.525924987	9.58E-06	485,241
ENSG00000198356	ASNA1	0.447441419	1.71E-05	271,984
ENSG00000169682	SPNS1	0.896876564	2.05E-05	677,032
ENSG00000213614	HEXA	−0.448754497	2.14E-05	52,058
ENSG00000103043	VAC14	0.401200129	2.46E-05	65,164 (45,876)

a Differentially expressed genes calculated by DESeq2. Cutoff *P* < 0.05.

b Distance from gene TSS to nearest ENCODE ZNF507 ChIP-seq peak. To account for additional peaks that would likely not be detected due to mapability, distances were also calculated for FIMO-predicted ZNF507 binding motifs in LINE-1 PA1, PA2, PA3, and PA4 subfamilies. When this distance was smaller it is indicated in parentheses.

### Identification of a physical interaction between ZNF507 and PRMT5

**Fig. 7.**
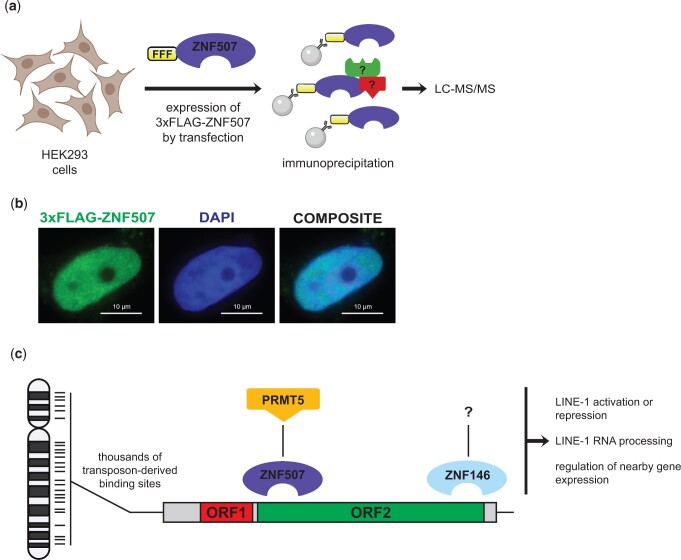
Identification of an interaction between ZNF507 and PMRT5. a) Schematic of approach to identify ZNF507-interacting proteins. HEK293 cells were transiently transfected with an 3xFLAG tagged ZNF507 expression plasmid or an empty vector control. After 48 h proteins were extracted and subjected to immunoprecipitation using anti-FLAG beads. Proteins were eluted by competition with 3xFLAG peptide before limited short gel electrophoresis and analysis by mass spectrometry (LC-MS/MS). b) Immunofluorescence analysis of HEK293 cells expressing 3xFLAG-ZNF507. All interphase cells observed expressing 3xFLAG-ZNF507 had nuclear localization. No appreciable signal was observed for cells transfected with empty vector 3xFLAG control plasmid. c) Model for LINE-1 binding by ZNF146 and ZNF507 and potential functions. ZNF146 and ZNF507 bind at thousands of sites in the genome as a result of LINE-1 transposition. Binding by either protein may then recruit various activities to these regions, such as the potential for ZNF507 to recruit protein arginine methylation activity through interaction with PRMT5. These activities may then contribute to context or cell-type dependent regulation of LINE-1 elements or nearby genes. Schematics created with BioRender.com.

Transcription factors frequently regulate gene expression by recruiting chromatin modifying complexes to regulatory sequences. Hence, we wondered whether ZNF507 might associate with a known chromatin modifying enzyme. To test this idea, we expressed 3xFLAG-tagged ZNF507 in HEK293 cells by transient transfection (Fig. 7a) and performed co-immunoprecipitation followed by unbiased interaction discovery by liquid chromatography with tandem mass spectrometry (LC-MS/MS). FLAG-tagged ZNF507 localized as expected to the nucleus in HEK293 cells (Fig. 7b). Immunoprecipitation (IP) was performed on cells expressing 3xFLAG-ZNF507 as well as an empty vector control using anti-FLAG beads. Proteins were eluted with 3xFLAG peptide and partially resolved by SDS-PAGE prior to proteomic analysis by LC-MS/MS.

As expected, bait protein, ZNF507, had the highest peptide count in the 3xFLAG-ZNF507 IP with far more peptides in this sample compared to empty vector control (Table 2). The second most abundant protein (by peptide count) was protein arginine methyltransferase 5 (PRMT5), again with far more peptides detected in the 3xFLAG-ZNF507 IP than for the EV control. Interestingly, PRMT5 is a known transcriptional and post-transcriptional regulator of gene expression with various protein targets including core histones (Dacwag *et al.* 2007; Lacroix *et al.* 2008; Bedford and Clarke 2009; Tee *et al.* 2010; LeBlanc *et al.* 2016). These results suggest ZNF507 physically associates with PRMT5 and may recruit arginine methylation to L1 sequences to regulate L1 or endogenous gene expression (see model, Fig. 7c).

**Table 2.** Results of co-IP LC-MS/MS.

	**No. of peptides** ^a^	
Protein	3xFLAG-ZNF507	EV
ZNF507	285	11
PRMT5	45	12
VCP	36	5
PPM1B	35	11
JUP	33	14
PRKDC	33	0
ANKFY1	28	5
DSG1	23	10
EEF1A1	22	5
SLC25A5	22	1
STK38	21	5
EIF4B	20	4
RBM10	20	5
AMY1A	19	8
EEF1G	19	3
ROCK1	19	1
POF1B	19	1
LMNA	18	0
ACAP2	17	1
WDR77	16	13
ANXA1	16	12
ANXA2	16	7
EIF3B	16	7
KCTD17	16	5
ENO1	16	1

a Results represent the most abundant proteins (by number of peptides observed) after LC-MS/MS analysis of immunoprecipitations from HEK293 cells transfected with either 3xFLAG-ZNF507 or EV control expression plasmids as indicated. Common contaminants: keratins, heat shock proteins, tubulins, DSPN, FLNA, TBB5, T complex were excluded from this table.

## Discussion

About half of the human genome is comprised of repeat sequences that may serve underexplored roles in coordinated genome regulation. Unearthing proteins that target repetitive sequences may therefore serve as a launching pad for greater understanding of such processes. Over the past decade thousands of next generation data sets have been produced and made publicly available, most of which were processed without repeat sequences in mind. Taking a repeat-centric approach to mining these data sets may facilitate better understanding of the repeat genome and its influence on the biology of higher organisms.

There are many potential approaches to studying repeat enrichment in next generation sequencing data sets (Teissandier *et al.* 2019). Here, we took a simple peak intersection approach and analyzed publish ENCODE data sets to identify proteins specifically enriched on repetitive transposon-derived sequences. We identified over 200 TF ChIP-seq peak sets enriched on specific transposon sequences more than would be expected by random chance. Strikingly, many DNA-binding proteins appear to specifically target ERV-K transposons, for reasons we cannot readily explain. We then focused on a prominent enrichment of 2 putative L1-targeting proteins, ZNF146/OZF and ZNF507, and characterized their relationship to L1 sequences.

Other groups have previously taken similar approaches to identify proteins enriched on repetitive elements in ChIP-seq data sets, either using related peak intersection approaches or taking additional steps to extract information from reads mapping to multiple places in the genome (Criscione *et al.* 2014; Jacobs *et al.* 2014; Schmitges *et al.* 2016; Barazandeh *et al.* 2018; Soraya Shehata 2020). Schmitges *et al.* used a similar approach to that taken here to calculate the percentage of peaks for ENCODE ChIP-seq data sets with TEs and independently identified ZNF146 as being enriched on L1 elements. During the preparation of this manuscript, ZNF507 was also recently identified by Shehata *et al.* as binding at L1 elements while performing large-scale analysis of ChIP-seq data sets.

In comparison to some other studies, particularly Barazandeh *et al.* and Schmitges *et al.* referenced above using similar data sets, relatively few DNA binding proteins were found to specifically recognize L1 repeats. These studies took a slightly different approach, using simple peak intersection frequencies rather than enrichment over shuffled background models to identify interactors. This difference likely caused our approach to under identify genuine TE-targeting proteins and highlight only those with high-frequency binding in the genome. Less frequent binders (for instance those that bind in the often-truncated 5’-end of L1 elements) would likely be missed by the approach taken here as these peak interactions would fail to enrich over background shuffling models when compared to high-abundance and highly fragmented elements such as L1.

Here, we focus on 2 such proteins, ZNF146 and ZNF507 targeting to L1 sequences and find these proteins target different regions of the ORF2-encoding region of L1 DNA sequences in the human genome. ZNF146 targets both “old” and “young” L1 near the extreme 3’ end of ORF2. In contrast, ZNF507 was found to bind the 5’ end of L1 ORF2 and was only observed at younger elements. Thus, our findings add to the knowledge base of proteins that specifically recognize L1 elements in the genome including other L1-targeting proteins shown to differentiate between L1 subfamilies (Jacobs *et al.* 2014). We show that changes in the amino acid composition of ZNF507 zinc fingers during primate evolution paralleled changes to L1 DNA sequence, indicating ZNF507 and L1 sequence may have co-evolved during this time period or perhaps that ZNF507 may have recently acquired affinity for L1 repeats.

Surprisingly little is known about the functions of either ZNF146/OZF or ZNF507 despite being widely expressed in human tissue (Uhlen *et al.* 2015, 2017). Several years ago ZNF146 was shown to be overexpressed in various cancers, though the contribution or molecular mechanisms related to ZNF146/OZF in oncogenic transformation remains opaque (Ferbus *et al.* 1999, 2003; Antoine *et al.* 2005b; Ma *et al.* 2020; Zhu *et al.* 2021). ZNF146 was shown to associate with telomeric protein Rap1 and thus may have a role in telomere maintenance (Antoine *et al.* 2005a). Recently, ZNF146 was additionally shown to associate with replisome subunits and influence replication fork progression under conditions of DNA replication stress (Feu *et al.* 2020). Future studies are needed to determine how LINE-1 targeting and these potential roles in regulating genomic integrity at telomeres and during replication are related.

Although we did not observe broad upregulation of L1 elements or identify any gene expression changes in HEK293 cells lacking ZNF146, it remains possible that ZNF146 has important roles for regulating transcription or epigenetic modifications in heterochromatic regions, which are enriched for L1 sequences. If so, their contribution to regulation may be obscured by DNA methylation or other redundant silencing mechanisms, analogous to the maintenance of inactive-X heterochromatin despite removal of XIST RNA (Csankovszki *et al.* 1999). Similarly, ZNF146 could have a more essential role in specific developmental states, as it was recently identified as a potential regulator of the naïve primate pluripotency network (Boroviak *et al.* 2018). Since LINE-1 elements are known to be activated and regulate chromatin dynamics during early embryogenesis (Fadloun *et al.* 2013; Jachowicz *et al.* 2017), it’s tempting to speculate ZNF146/OZF is either involved in this activation or important for subsequent repression of LINE-1 elements post-activation. Future studies may address these potential connections between ZNF146, LINE-1 elements, pluripotency, and early development.

ZNF507 has been identified in genomic studies as being a potential risk locus for neural developmental disorders (Talkowski *et al.* 2012; Curtis and UK10K Consortium 2016). Despite this potential relationship, to our knowledge ZNF507 had not been the subject of directed investigation. Our experiments here demonstrate ZNF507 targets L1 “young” elements and indicate ZNF507 depletion influences gene expression, potentially through interaction with PRMT5. Our transcriptomic analysis was performed in an embryonic kidney cell line (HEK293), which may not be the most relevant cell type to study ZNF507 function. Future experiments depleting ZNF507 in relevant neural cell types or neural organoids are needed to determine whether ZNF507 acts as a transcriptional regulator in these contexts. Identifying these targets could be key in determining what impact, if any, ZNF507 has on various brain disorders.

PRMT5 was recently shown to be a critical repressor of LINE-1 elements in primordial germ cells and early embryonic development (Kim *et al.* 2014). In this context, PRMT5-mediated methylation of histones is required to repress L1 expression during this unusual period of global reduction in DNA methylation. To our knowledge, how PRMT5 is targeted to LINE-1 sequences is largely unknown. Here, we identify a potential interaction between PRMT5 and ZNF507. Although this relationship requires further validation, results here indicate ZNF507’s primary function may be to recruit PRMT5-mediated arginine methyltransferase activity to chromatin in order to regulate L1 or endogenous gene expression during development.

## Data availability

Strains and plasmids are available upon request. Gene expression data are available at GEO with the accession number: GSE172285.

Supplementary material is available at *G3* online.

## Supplementary Material

jkac002_Supplementary_Data

## References

[jkac002-B1] Antoine K , Ferbus D, Kolahgar G, Prosperi MT, Goubin G. Zinc finger protein overexpressed in colon carcinoma interacts with the telomeric protein hRap1. J Cell Biochem. 2005a;95(4):763–768. 15838871 10.1002/jcb.20487

[jkac002-B2] Antoine K , Prosperi MT, Ferbus D, Boule C, Goubin G. A Kruppel zinc finger of ZNF 146 interacts with the SUMO-1 conjugating enzyme UBC9 and is sumoylated in vivo. Mol Cell Biochem. 2005b;271(1–2):215–223. 15881673 10.1007/s11010-005-6417-2

[jkac002-B3] Bailey TL , Elkan C. Fitting a mixture model by expectation maximization to discover motifs in biopolymers. Proc Int Conf Intell Syst Mol Biol. 1994;2:28–36. 7584402

[jkac002-B4] Barazandeh M , Lambert SA, Albu M, Hughes TR. Comparison of ChIP-Seq data and a reference motif set for human KRAB C2H2 zinc finger proteins. G3 (Bethesda). 2018;8(1):219–229. 29146583 10.1534/g3.117.300296 PMC5765350

[jkac002-B5] Bedford MT , Clarke SG. Protein arginine methylation in mammals: who, what, and why. Mol Cell. 2009;33(1):1–13. 19150423 10.1016/j.molcel.2008.12.013 PMC3372459

[jkac002-B6] Boroviak T , Stirparo GG, Dietmann S, Hernando-Herraez I, Mohammed H, Reik W, Smith A, Sasaki E, Nichols J, Bertone P. Single cell transcriptome analysis of human, marmoset and mouse embryos reveals common and divergent features of preimplantation development. Development. 2018;145(21):dev167833. 30413530 10.1242/dev.167833 PMC6240320

[jkac002-B7] Bourque G , Leong B, Vega VB, Chen X, Lee YL, Srinivasan KG, Chew JL, Ruan Y, Wei CL, Ng HH, et al Evolution of the mammalian transcription factor binding repertoire via transposable elements. Genome Res. 2008;18(11):1752–1762. 18682548 10.1101/gr.080663.108 PMC2577865

[jkac002-B8] Brouha B , Schustak J, Badge RM, Lutz-Prigge S, Farley AH, Moran JV, Kazazian HH. Jr., Hot L1s account for the bulk of retrotransposition in the human population. Proc Natl Acad Sci USA. 2003;100(9):5280–5285. 12682288 10.1073/pnas.0831042100 PMC154336

[jkac002-B9] Burns KH. Transposable elements in cancer. Nat Rev Cancer. 2017;17(7):415–424. 28642606 10.1038/nrc.2017.35

[jkac002-B10] Cosby RL , Chang NC, Feschotte C. Host-transposon interactions: conflict, cooperation, and cooption. Genes Dev. 2019;33(17–18):1098–1116. 31481535 10.1101/gad.327312.119 PMC6719617

[jkac002-B11] Coufal NG , Garcia-Perez JL, Peng GE, Yeo GW, Mu Y, Lovci MT, Morell M, O'Shea KS, Moran JV, Gage FH. L1 retrotransposition in human neural progenitor cells. Nature. 2009;460(7259):1127–1131. 19657334 10.1038/nature08248 PMC2909034

[jkac002-B12] Criscione SW , Zhang Y, Thompson W, Sedivy JM, Neretti N. Transcriptional landscape of repetitive elements in normal and cancer human cells. BMC Genomics. 2014;15:583. 25012247 10.1186/1471-2164-15-583 PMC4122776

[jkac002-B13] Csankovszki G , Panning B, Bates B, Pehrson JR, Jaenisch R. Conditional deletion of Xist disrupts histone macroH2A localization but not maintenance of X inactivation. Nat Genet. 1999;22(4):323–324. 10431231 10.1038/11887

[jkac002-B14] Curtis D ; UK10K Consortium. Practical experience of the application of a weighted burden test to whole exome sequence data for obesity and schizophrenia. Ann Hum Genet. 2016;80(1):38–49. 26474449 10.1111/ahg.12135 PMC4833177

[jkac002-B15] Dacwag CS , Ohkawa Y, Pal S, Sif S, Imbalzano AN. The protein arginine methyltransferase Prmt5 is required for myogenesis because it facilitates ATP-dependent chromatin remodeling. Mol Cell Biol. 2007;27(1):384–394. 17043109 10.1128/MCB.01528-06 PMC1800640

[jkac002-B16] Deininger P , Morales ME, White TB, Baddoo M, Hedges DJ, Servant G, Srivastav S, Smither ME, Concha M, DeHaro DL, et al A comprehensive approach to expression of L1 loci. Nucleic Acids Res. 2017;45(5):e31. 27899577 10.1093/nar/gkw1067 PMC5389711

[jkac002-B17] Dewannieux M , Esnault C, Heidmann T. LINE-mediated retrotransposition of marked Alu sequences. Nat Genet. 2003;35(1):41–48. 12897783 10.1038/ng1223

[jkac002-B18] ENCODE Project Consortium. An integrated encyclopedia of DNA elements in the human genome. Nature. 2012;489:57–74. 22955616 10.1038/nature11247 PMC3439153

[jkac002-B19] Ewing AD , Kazazian HH Jr. High-throughput sequencing reveals extensive variation in human-specific L1 content in individual human genomes. Genome Res. 2010;20(9):1262–1270. 20488934 10.1101/gr.106419.110 PMC2928504

[jkac002-B20] Fadloun A , Le Gras S, Jost B, Ziegler-Birling C, Takahashi H, Gorab E, Carninci P, Torres-Padilla ME. Chromatin signatures and retrotransposon profiling in mouse embryos reveal regulation of LINE-1 by RNA. Nat Struct Mol Biol. 2013;20(3):332–338. 23353788 10.1038/nsmb.2495

[jkac002-B21] Faulkner GJ , Garcia-Perez JL. L1 Mosaicism in mammals: extent, effects, and eEvolution. Trends Genet. 2017;33(11):802–816. 28797643 10.1016/j.tig.2017.07.004

[jkac002-B22] Ferbus D , Antoine K, Goubin G. Production and characterization of mouse monoclonal antibodies to human zinc finger OZF protein overexpressed in pancreatic carcinomas. Hybridoma. 1999;18(5):431–436. 10600030 10.1089/hyb.1999.18.431

[jkac002-B23] Ferbus D , Bovin C, Validire P, Goubin G. The zinc finger protein OZF (ZNF146) is overexpressed in colorectal cancer. J Pathol. 2003;200(2):177–182. 12754738 10.1002/path.1337

[jkac002-B24] Feu S , Unzueta F, Llopis A, Semple JI, Ercilla A, Guaita-Esteruelas S, Jaumot M, Freire R, Agell N. OZF is a Claspin-interacting protein essential to maintain the replication fork progression rate under replication stress. FASEB J. 2020;34(5):6907–6919. 32267586 10.1096/fj.201901926R

[jkac002-B25] Giordano J , Ge Y, Gelfand Y, Abrusan G, Benson G, Warburton PE. Evolutionary history of mammalian transposons determined by genome-wide defragmentation. PLoS Comput Biol. 2007;3(7):e137. 17630829 10.1371/journal.pcbi.0030137 PMC1914374

[jkac002-B26] Grant CE , Bailey TL, Noble WS. FIMO: scanning for occurrences of a given motif. Bioinformatics. 2011;27(7):1017–1018. 21330290 10.1093/bioinformatics/btr064 PMC3065696

[jkac002-B27] Hall LL , Carone DM, Gomez AV, Kolpa HJ, Byron M, Mehta N, Fackelmayer FO, Lawrence JB. Stable C0T-1 repeat RNA is abundant and is associated with euchromatic interphase chromosomes. Cell. 2014;156(5):907–919. 24581492 10.1016/j.cell.2014.01.042 PMC4023122

[jkac002-B28] Imbeault M , Helleboid PY, Trono D. KRAB zinc-finger proteins contribute to the evolution of gene regulatory networks. Nature. 2017;543(7646):550–554. 28273063 10.1038/nature21683

[jkac002-B29] Jachowicz JW , Bing X, Pontabry J, Boskovic A, Rando OJ, Torres-Padilla ME. LINE-1 activation after fertilization regulates global chromatin accessibility in the early mouse embryo. Nat Genet. 2017;49(10):1502–1510. 28846101 10.1038/ng.3945

[jkac002-B30] Jacobs FM , Greenberg D, Nguyen N, Haeussler M, Ewing AD, Katzman S, Paten B, Salama SR, Haussler D. An evolutionary arms race between KRAB zinc-finger genes ZNF91/93 and SVA/L1 retrotransposons. Nature. 2014;516(7530):242–245. 25274305 10.1038/nature13760 PMC4268317

[jkac002-B31] Kazazian HH Jr , Moran JV. Mobile DNA in health and disease. N Engl J Med. 2017;377(4):361–370. 28745987 10.1056/NEJMra1510092 PMC5980640

[jkac002-B32] Khan H , Smit A, Boissinot S. Molecular evolution and tempo of amplification of human LINE-1 retrotransposons since the origin of primates. Genome Res. 2006;16(1):78–87. 16344559 10.1101/gr.4001406 PMC1356131

[jkac002-B33] Kigami D , Minami N, Takayama H, Imai H. MuERV-L is one of the earliest transcribed genes in mouse one-cell embryos. Biol Reprod. 2003;68(2):651–654. 12533431 10.1095/biolreprod.102.007906

[jkac002-B34] Kim S , Gunesdogan U, Zylicz JJ, Hackett JA, Cougot D, Bao S, Lee C, Dietmann S, Allen GE, Sengupta R, et al PRMT5 protects genomic integrity during global DNA demethylation in primordial germ cells and preimplantation embryos. Mol Cell. 2014;56(4):564–579. 25457166 10.1016/j.molcel.2014.10.003 PMC4250265

[jkac002-B35] Kolosha VO , Martin SL. In vitro properties of the first ORF protein from mouse LINE-1 support its role in ribonucleoprotein particle formation during retrotransposition. Proc Natl Acad Sci USA. 1997;94(19):10155–10160. 9294179 10.1073/pnas.94.19.10155 PMC23331

[jkac002-B36] Konkel MK , Walker JA, Batzer MA. LINEs and SINEs of primate evolution. Evol Anthropol. 2010;19(6):236–249. 25147443 10.1002/evan.20283 PMC4137791

[jkac002-B37] Korenberg JR , Rykowski MC. Human genome organization: alu, lines, and the molecular structure of metaphase chromosome bands. Cell. 1988;53(3):391–400. 3365767 10.1016/0092-8674(88)90159-6

[jkac002-B38] Lacroix M , El Messaoudi S, Rodier G, Le Cam A, Sardet C, Fabbrizio E. The histone-binding protein COPR5 is required for nuclear functions of the protein arginine methyltransferase PRMT5. EMBO Rep. 2008;9(5):452–458. 18404153 10.1038/embor.2008.45 PMC2373370

[jkac002-B39] Laity JH , Lee BM, Wright PE. Zinc finger proteins: new insights into structural and functional diversity. Curr Opin Struct Biol. 2001;11(1):39–46. 11179890 10.1016/s0959-440x(00)00167-6

[jkac002-B40] LeBlanc SE , Wu Q, Lamba P, Sif S, Imbalzano AN. Promoter-enhancer looping at the PPARgamma2 locus during adipogenic differentiation requires the Prmt5 methyltransferase. Nucleic Acids Res. 2016;44(11):5133–5147. 26935580 10.1093/nar/gkw129 PMC4914087

[jkac002-B41] Li H , Durbin R. Fast and accurate short read alignment with Burrows-Wheeler transform. Bioinformatics. 2009;25(14):1754–1760. 19451168 10.1093/bioinformatics/btp324 PMC2705234

[jkac002-B42] Liao Y , Smyth GK, Shi W. featureCounts: an efficient general purpose program for assigning sequence reads to genomic features. Bioinformatics. 2014;30(7):923–930. 24227677 10.1093/bioinformatics/btt656

[jkac002-B43] Lu JY , Chang L, Li T, Wang T, Yin Y, Zhan G, Han X, Zhang K, Tao Y, Percharde M, et al Homotypic clustering of L1 and B1/Alu repeats compartmentalizes the 3D genome. Cell Res. 2021;31(6):613–630. 33514913 10.1038/s41422-020-00466-6 PMC8169921

[jkac002-B44] Ma Y , Cong X, Zhang Y, Yin X, Zhu Z, Xue Y. CircPIP5K1A facilitates gastric cancer progression via miR-376c-3p/ZNF146 axis. Cancer Cell Int. 2020;20:81. 32190005 10.1186/s12935-020-1122-5 PMC7071687

[jkac002-B45] Manuelidis L , Ward DC. Chromosomal and nuclear distribution of the HindIII 1.9-kb human DNA repeat segment. Chromosoma. 1984;91(1):28–38. 6098426 10.1007/BF00286482

[jkac002-B46] Mathias SL , Scott AF, Kazazian HH Jr, Boeke JD, Gabriel A. Reverse transcriptase encoded by a human transposable element. Science. 1991;254(5039):1808–1810. 1722352 10.1126/science.1722352

[jkac002-B47] Mir AA , Philippe C, Cristofari G. euL1db: the European database of L1HS retrotransposon insertions in humans. Nucleic Acids Res. 2015;43:D43–D47. 25352549 10.1093/nar/gku1043 PMC4383891

[jkac002-B48] Moran JV , Holmes SE, Naas TP, DeBerardinis RJ, Boeke JD, Kazazian HH Jr. High frequency retrotransposition in cultured mammalian cells. Cell. 1996;87(5):917–927. 8945518 10.1016/s0092-8674(00)81998-4

[jkac002-B49] Ostertag EM , Goodier JL, Zhang Y, Kazazian HH Jr. SVA elements are nonautonomous retrotransposons that cause disease in humans. Am J Hum Genet. 2003;73(6):1444–1451. 14628287 10.1086/380207 PMC1180407

[jkac002-B50] Penzkofer T , Jager M, Figlerowicz M, Badge R, Mundlos S, Robinson PN, Zemojtel T. L1Base 2: more retrotransposition-active LINE-1s, more mammalian genomes. Nucleic Acids Res. 2017;45(D1):D68–D73. 27924012 10.1093/nar/gkw925 PMC5210629

[jkac002-B51] Persikov AV , Osada R, Singh M. Predicting DNA recognition by Cys2His2 zinc finger proteins. Bioinformatics. 2009;25(1):22–29. 19008249 10.1093/bioinformatics/btn580 PMC2638941

[jkac002-B52] Philippe C , Vargas-Landin DB, Doucet AJ, van Essen D, Vera-Otarola J, Kuciak M, Corbin A, Nigumann P, Cristofari G. Activation of individual L1 retrotransposon instances is restricted to cell-type dependent permissive loci. Elife. 2016;5:e13926. 27016617 10.7554/eLife.13926 PMC4866827

[jkac002-B53] Quinlan AR , Hall IM. BEDTools: a flexible suite of utilities for comparing genomic features. Bioinformatics. 2010;26(6):841–842. 20110278 10.1093/bioinformatics/btq033 PMC2832824

[jkac002-B54] Ramirez F , Dundar F, Diehl S, Gruning BA, Manke T. deepTools: a flexible platform for exploring deep-sequencing data. Nucleic Acids Res. 2014;42:W187–W191. 24799436 10.1093/nar/gku365 PMC4086134

[jkac002-B55] Ran FA , Hsu PD, Wright J, Agarwala V, Scott DA, Zhang F. Genome engineering using the CRISPR-Cas9 system. Nat Protoc. 2013;8(11):2281–2308. 24157548 10.1038/nprot.2013.143 PMC3969860

[jkac002-B56] Rebollo R , Romanish MT, Mager DL. Transposable elements: an abundant and natural source of regulatory sequences for host genes. Annu Rev Genet. 2012;46:21–42. 22905872 10.1146/annurev-genet-110711-155621

[jkac002-B57] Robbez-Masson L , Rowe HM. Retrotransposons shape species-specific embryonic stem cell gene expression. Retrovirology. 2015;12:45. 26021318 10.1186/s12977-015-0173-5 PMC4448215

[jkac002-B58] Schmitges FW , Radovani E, Najafabadi HS, Barazandeh M, Campitelli LF, Yin Y, Jolma A, Zhong G, Guo H, Kanagalingam T, et al Multiparameter functional diversity of human C2H2 zinc finger proteins. Genome Res. 2016;26(12):1742–1752. 27852650 10.1101/gr.209643.116 PMC5131825

[jkac002-B59] Smit AF , Toth G, Riggs AD, Jurka J. Ancestral, mammalian-wide subfamilies of LINE-1 repetitive sequences. J Mol Biol. 1995;246(3):401–417. 7877164 10.1006/jmbi.1994.0095

[jkac002-B60] Soraya Shehata SS , Swearingen A, Wheeler G, Das A, Corbet G, Nebenfuehr B, Ahrens D, Tauber D, Lennon S, Choi K, et al Genome-wide binding analysis of 195 DNA Binding Proteins reveals “reservoir” promoters and human specific SVA-repeat family regulation. Biorxiv. 2020. 10.1101/2020.07.21.213603. PMC8224974 34166368

[jkac002-B61] Talkowski ME , Rosenfeld JA, Blumenthal I, Pillalamarri V, Chiang C, Heilbut A, Ernst C, Hanscom C, Rossin E, Lindgren AM, et al Sequencing chromosomal abnormalities reveals neurodevelopmental loci that confer risk across diagnostic boundaries. Cell. 2012;149(3):525–537. 22521361 10.1016/j.cell.2012.03.028 PMC3340505

[jkac002-B62] Tee WW , Pardo M, Theunissen TW, Yu L, Choudhary JS, Hajkova P, Surani MA. Prmt5 is essential for early mouse development and acts in the cytoplasm to maintain ES cell pluripotency. Genes Dev. 2010;24(24):2772–2777. 21159818 10.1101/gad.606110 PMC3003195

[jkac002-B63] Teissandier A , Servant N, Barillot E, Bourc'his D. Tools and best practices for retrotransposon analysis using high-throughput sequencing data. Mob DNA. 2019;10:52. 31890048 10.1186/s13100-019-0192-1 PMC6935493

[jkac002-B64] Uhlen M , Fagerberg L, Hallstrom BM, Lindskog C, Oksvold P, Mardinoglu A, Sivertsson A, Kampf C, Sjostedt E, Asplund A, et al Proteomics. Tissue-based map of the human proteome. Science. 2015;347(6220):1260419. 25613900 10.1126/science.1260419

[jkac002-B65] Uhlen M , Zhang C, Lee S, Sjostedt E, Fagerberg L, Bidkhori G, Benfeitas R, Arif M, Liu Z, Edfors F, et al A pathology atlas of the human cancer transcriptome. Science. 2017;357(6352):eaan2507. 28818916 10.1126/science.aan2507

[jkac002-B66] Vijayabaskar MS , Goode DK, Obier N, Lichtinger M, Emmett AML, Abidin FNZ, Shar N, Hannah R, Assi SA, Lie ALM, et al Identification of gene specific cis-regulatory elements during differentiation of mouse embryonic stem cells: an integrative approach using high-throughput datasets. PLoS Comput Biol. 2019;15(11):e1007337. 31682597 10.1371/journal.pcbi.1007337 PMC6855567

[jkac002-B67] Wang H , Xing J, Grover D, Hedges DJ, Han K, Walker JA, Batzer MA. SVA elements: a hominid-specific retroposon family. J Mol Biol. 2005;354(4):994–1007. 16288912 10.1016/j.jmb.2005.09.085

[jkac002-B68] Wang J , Xie G, Singh M, Ghanbarian AT, Rasko T, Szvetnik A, Cai H, Besser D, Prigione A, Fuchs NV, et al Primate-specific endogenous retrovirus-driven transcription defines naive-like stem cells. Nature. 2014;516(7531):405–409. 25317556 10.1038/nature13804

[jkac002-B69] Yang F , Wang PJ. Multiple LINEs of retrotransposon silencing mechanisms in the mammalian germline. Semin Cell Dev Biol. 2016;59:118–125. 26957474 10.1016/j.semcdb.2016.03.001 PMC5011444

[jkac002-B70] Yang P , Wang Y, Macfarlan TS. The role of KRAB-ZFPs in transposable element repression and mammalian evolution. Trends Genet. 2017;33(11):871–881. 28935117 10.1016/j.tig.2017.08.006 PMC5659910

[jkac002-B71] Zhu S , Chen CY, Hao Y. LncRNA KCNQ1OT1 acts as miR-216b-5p sponge to promote colorectal cancer progression via up-regulating ZNF146. J Mol Histol. 2021;52(3):479–490. 33394291 10.1007/s10735-020-09942-0

